# Combined efficacy of C-reactive protein and red blood cell distribution width in prognosis of patients with culture-negative infective endocarditis

**DOI:** 10.18632/oncotarget.16888

**Published:** 2017-04-06

**Authors:** Xue-biao Wei, Yuan-hui Liu, Peng-cheng He, Ying-ling Zhou, Ning Tan, Ji-yan Chen, Dan-qing Yu

**Affiliations:** ^1^ Department of Cardiology, Guangdong Cardiovascular Institute, Guangdong Provincial Key Laboratory of Coronary Heart Disease Prevention, Guangdong General Hospital, Guangdong Academy of Medical Sciences, Guangzhou 510100, Guangdong, China

**Keywords:** infective endocarditis, c-reactive protein, red blood cell distribution width, outcome

## Abstract

**Objective:**

To evaluate the combined effect of C-reactive protein (CRP) and red blood cell distribution width (RDW) on the prediction of in-hospital and long-term poor outcomes in patients with blood culture-negative infective endocarditis (BCNE).

**Results:**

Patients with high CRP and high RDW has the highest incidence of in-hospital death (2.3% vs. 7.8% vs. 5.6% vs. 17.5%, *P* < 0.001). CRP > 17.8 mg/L (odds ratio [OR]=2.41, 95% confidence interval [CI], 1.06–5.51, *P =* 0.037), RDW >16.3 (OR = 2.29, 95% CI, 1.10–4.77, *P =* 0.027), and these two values in combination (OR = 3.15, 95% CI, 1.46–6.78, P=0.003) were independently associated with in-hospital death. Patients with RDW > 16.3 had higher long-term mortality (*P* = 0.003), while no significant correlation was observed for CRP (*P =* 0.151).

**Materials and Methods:**

In total, 572 participants with BCNE were consecutively enrolled. They were classified into four groups based on the optimal CRP and RDW cut-off values (which were determined using a receiver operating characteristic analysis): low CRP and low RDW (*n =* 216), high CRP and low RDW (*n =* 129), low CRP and high RDW (*n =* 107), and high CRP and high RDW (*n =* 120).

**Conclusions:**

Increased CRP and RDW, especially in combination, are independently associated with in-hospital death in BCNE. RDW, but not CRP, has long-term prognostic value.

## INTRODUCTION

Despite intensive antibiotic therapy and aggressive surgical intervention, infective endocarditis (IE) remains a serious and potentially life-threatening condition [1]. The mortality rate of IE is about 20% after initial admission and up to 40% after 5 years [2–4]. Early detection of high-risk patients is essential for improving prognosis. Furthermore, among IE patients, 5–69.7% are blood culture-negative with relatively poor outcomes, particularly in developing countries [5–7]. However, limited studies to date have focused on this patient population.

Several factors, including old age, contribute to high mortality and morbidity rates in IE patients [8–10]. In addition, elevation of C-reactive protein (CRP) and red blood cell distribution width (RDW) have been shown to be associated, at least partly, with poor outcomes in IE patients [11–14]. Nevertheless, the results in the literature to date are inconclusive, and numerous factors, such as different investigation protocols and study sample sizes, have been suggested to explain these discrepancies. The present study was aimed at evaluating the predictive value of combined CRP and RDW for outcomes of patients with blood culture-negative infective endocarditis (BCNE).

## RESULTS

### Baseline clinical characteristics based on CRP and RDW

**Figure 1:**
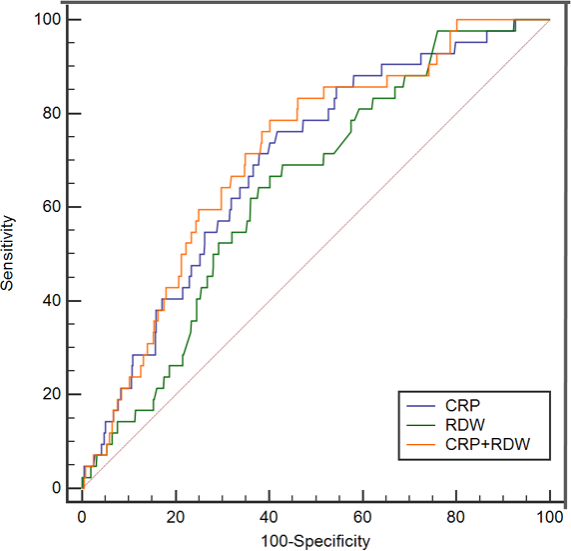
The ROC curves for CRP and RDW in predicting in-hospital death

In-hospital death was recorded for 42 of the 572 patients (72.0% men aged 45 ± 15 years, range, 18–83 years) definitely diagnosed as having BCNE. In the ROC analysis, CRP > 17.8 mg/L showed a sensitivity of 76.2% and a specificity of 58.3% for predicting in-hospital death (AUC = 0.696, 95% CI, 0.620–0.772, *P <* 0.001). RDW > 16.3% showed a sensitivity of 64.3% and a specificity of 62.3% (AUC = 0.641, 95% CI, 0.564–0.718, *P =* 0.002). We observed no significant differences between CRP and RDW in predicting in-hospital death (AUC: 0.696 vs. 0.641, *P =* 0.308; Figure 1). AUC for combined CRP and RDW was 0.712 (95% CI, 0.638–0.785, *P <* 0.001) with a sensitivity of 78.6% and specificity of 59.8%, and did not achieve better performance than RDW only (AUC: 0.712 vs. 0.641, *P =* 0.056).

Patients were divided into four groups according to the optimal cut-off values of CRP and RDW, specifically, 216 with low CRP and low RDW, 129 with high CRP and low RDW, 107 with low CRP and high RDW and 120 with high CRP and high RDW. Patients with low CRP and high RDW were more likely to be female and those with low CRP and low RDW to have a history of IE. In addition, lower rates of prior antibiotic use and New York Heart Association (NYHA) class III-IV were seen in patients with low CRP and low RDW. Higher levels of serum creatinine were detected in patients with high CRP and high RDW displaying lower concentrations of hemoglobin. Patients with high CRP were associated with higher erythrocyte sedimentation rate (ESR) and white blood count (WBC). Overall, 451 (78.8%) patients received surgical treatment, with the lowest rate recorded for the subgroup of patients with high CRP and high RDW (88.4% vs. 72.9% vs. 78.5% vs. 68.3%, *P <* 0.001). Moreover, in-hospital death rates (2.3% vs. 7.8% vs. 5.6% vs. 17.5%, *P <* 0.001) and MACEs (10.2% vs. 24.8% vs. 17.8% vs. 38.3%, *P <* 0.001) were significantly higher in patients with high CRP and high RDW, relative to the other subgroups. All surviving patients were followed up for a median of 32.4 (13.7–54.0) months (65 lost to follow-up). We observed statistical differences among the groups in terms of long-term mortality (4.9% vs. 6.7% vs. 10.2% vs. 14.9%, *P =* 0.032) (Table 1).

**Table 1:** Baseline clinical characteristics of patients according to CRP and RDW

Clinical variables	Low CRP, Low RDW (*n =* 216)	High CRP, Low RDW (*n =* 129)	Low CRP, High RDW (*n =* 107)	High CRP, High RDW (*n =* 120)	*P*-value
Age (year)	44.4 ± 13.9	47.1 ± 15.6	44.3 ± 14.5	43.4 ± 16.1	*0.227*
Males, *n* (%)	161 (74.5)	102 (79.1)	64 (59.8)	85 (70.8)	*0.008*
Hypertension, *n* (%)	34 (15.7)	20 (15.5)	9 (8.4)	15 (12.5)	0.283
Diabetes mellitus, *n* (%)	9 (4.2)	10 (7.8)	8 (7.5)	10 (8.3)	0.375
Previous IE	24 (11.1)	2 (1.6)	4 (3.7)	9 (7.5)	0.004
Prior antibiotic use	102 (47.2)	83 (64.3)	68 (63.6)	74 (61.7)	0.003
Prosthetic valves	9 (4.2)	9 (7.0)	8 (7.5)	6 (5.0)	0.552
Symptoms > 1 month	131 (60.6)	52 (40.3)	70 (65.4)	59 (49.2)	< 0.001
NYHA III–IV, *n* (%)	61 (28.2)	49 (38.0)	41 (38.3)	64 (53.3)	< 0.001
Affected valve					
Aortic Mitral Aortic+Mitral Others	75 (34.7) 89 (41.2) 13 (6.0) 22 (10.2)	51 (39.5) 49 (38.0) 10 (7.8) 7 (5.4)	36 (33.6) 40 (37.4) 11 (10.3) 14 (13.1)	37 (30.8) 51 (42.5) 17 (14.2) 13 (10.8)	0.538 0.809 0.080 0.233
Paravalvular abscess	7 (3.2)	12 (9.3)	6 (5.6)	8 (6.7)	0.128
CRP, mg/L	3.6 (1.9,7.5)	41.3 (27.7, 69.8)	7.7 (4.3, 12.9)	39.9 (26.5, 71.3)	< 0.001
ESR, mm/h	10.0 (4.0,28.0)	54.0 (26.0, 81.0)	25.0 (7.0, 51.3)	57.0 (28.0, 95.0)	< 0.001
Creatinine, umol/L	84.2 ± 26.7	83.4 ± 23.1	90.2 ± 48.9	98.7 ± 51.1	0.003
WBC (*10^9/L)	7.4 ± 2.7	10.9 ± 4.8	8.4 ± 3.3	11.7 ± 6.0	< 0.001
Hemoglobin, g/L	128.6 ± 20.6	109.4 ± 18.6	105.5 ± 21.3	95.3 ± 17.8	< 0.001
RDW (%)	14.2 ± 1.1	14.5 ± 1.0	19.1 ± 2.7	19.5 ± 3.2	< 0.001
LVEF (%)	65.0 ± 8.4	64.6 ± 8.5	63.8 ± 8.2	63.7 ± 9.7	0.539
Vegetation size > 10 mm	79 (36.6)	62 (48.1)	48 (44.9)	66 (55.0)	*0.009*
Surgical treatment	191 (88.4)	94 (72.9)	84 (78.5)	82 (68.3)	*< 0.001*
Type of surgery					
Bioprosthesis	30 (15.7)	11 (11.7)	18 (21.4)	12 (14.6)	
Mechanical prosthesis	131 (68.6)	70 (74.5)	58 (69.0)	62 (75.6)	
Repair	30 (15.7)	13 (13.8)	9 (9.5)	8 (9.8)	*0.432*
In-hospital events					
Embolic events Stroke Death MACEs	11 (5.1) 7 (3.2) 5 (2.3) 22 (10.2)	15 (11.6) 10 (7.8) 10 (7.8) 32 (24.8)	7 (6.5) 5 (4.7) 6 (5.6) 19 (17.8)	16 (13.3) 9 (7.5) 21 (17.5) 46 (38.3)	*0.030* *0.212* *< 0.001* *< 0.001*
Long-term mortality	9 (4.9)	7 (6.7)	9 (10.2)	13 (14.9)	*0.032*

Abbreviations: IE, infective endocarditis; NYHA, New York Heart Association; CRP, C-reactive protein; ESR, erythrocyte sedimentation rate; WBC,white blood count; RDW, red cell distribution width; LVEF, left ventricular ejection fraction; MACEs, major adverse clinical events.

Patients with high RDW were more likely to have a long disease course (symptoms > 1 month). Subgroup analysis for patients with symptoms for > 1 month or not was additionally performed. CRP was higher in the in-hospital death patient group, regardless of the duration of IE. However, differences in RDW were only observed in patients with symptoms ≤ 1 month.

### Predictive value of CRP and RDW for adverse outcomes

Univariate logistic regression identified age (odds ratio [OR] = 1.05, *P <* 0.001), WBC (OR = 1.06, *P =* 0.028), eGFR < 90 mL/min/1.73 m^2^ (OR = 3.26, *P =* 0.001), LVEF (OR = 0.95, *P =* 0.001), surgical treatment (OR = 0.12, *P <* 0.001), CRP (OR = 1.01, *P =* 0.001) and RDW (OR = 1.12, *P =* 0.007) as significant predictors for in-hospital death. In multivariate analysis, CRP > 17.8 mg/L (OR = 2.41, 95% CI, 1.06–5.51, *P =* 0.037) and RDW > 16.3 (OR = 2.29, 95% CI, 1.10–4.77, *P =* 0.027) were found to independently predict in-hospital mortality, with the combination of CRP and RDW being the strongest predictor (OR = 3.15, 95% CI, 1.46–6.78, *P =* 0.003). Other factors, including age, LVEF and surgical treatment, were significantly associated with in-hospital mortality (Table 2).

**Table 2:** Risk factors of in-hospital death by multivariate logistic regression

Clinical variables	Odds ratio	95% Confidence interval (CI)	*p*-value
**Model 1**			
Age (year)	1.03	1.01,1.06	0.021
WBC (*10^9^/L)	1.09	1.03,1.16	0.005
eGFR < 90 ml/min/1.73 m2	2.15	0.98,4.71	0.055
LVEF (%)	0.96	0.92,0.99	0.015
Surgical treatment	0.17	0.08,0.34	< 0.001
**Model 2**			
CRP > 17.8 mg/L	2.41	1.06,5.51	0.037
Age (year)	1.03	1.00,1.05	0.040
WBC (*10^9^/L)	1.07	1.00,1.14	0.061
eGFR < 90 ml/min/1.73 m2	2.14	0.97,4.70	0.059
LVEF (%)	0.96	0.93,1.00	0.032
Surgical treatment	0.20	0.10,0.41	< 0.001
**Model 3**			
RDW > 16.3%	2.29	1.10,4.77	0.027
Age (year)	1.03	1.01,1.06	0.015
WBC (*10^9^/L)	1.08	1.01,1.15	0.018
eGFR < 90 ml/min/1.73 m2	1.97	0.89,4.35	0.094
LVEF (%)	0.96	0.92,0.99	0.017
Surgical treatment	0.18	0.09,0.36	< 0.001
**Model 4**			
CRP > 17.8 mg/L and RDW > 16.3%	3.15	1.46,6.78	0.003
Age (year)	1.03	1.01,1.06	0.015
WBC (*10^9^/L)	1.06	0.99,1.13	0.090
eGFR < 90 ml/min/1.73 m2	1.95	0.88,4.32	0.098
LVEF (%)	0.96	0.93,1.00	0.044
Surgical treatment	0.19	0.09,0.39	< 0.001

Abbreviations: WBC, white blood count; eGFR, estimated glomerular filtration rate; LVEF, left ventricular ejection fraction; CRP, C-reactive protein; RDW, red cell distribution width; age, WBC and LVEF were continuous variables here.

**Figure 2:**
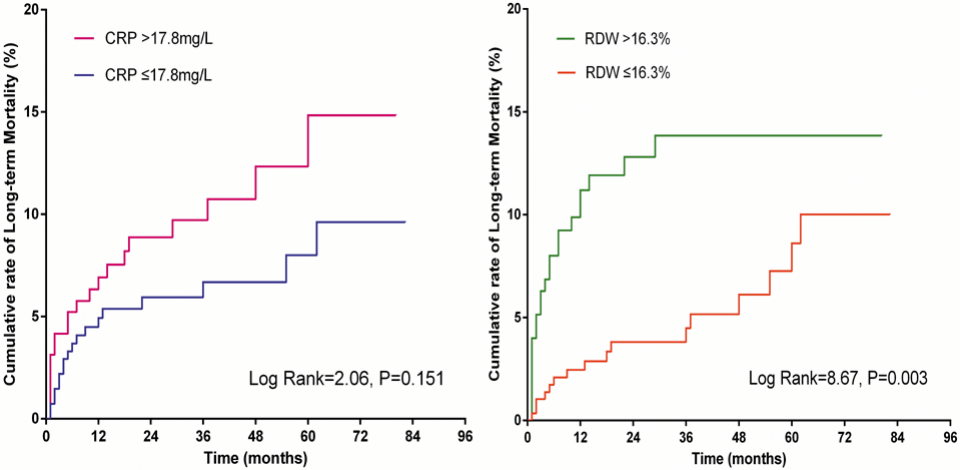
Kaplan-Meier curves of CRP and RDW for long-term mortality

Kaplan-Meier analysis showed a higher cumulative rate of long-term death in patients with RDW > 16.3 (log rank = 8.67, *P =* 0.003). However, this association was not observed for CRP (log rank = 2.06, *P =* 0.151; Figure 2).

## DISCUSSION

To our knowledge, the present study is the first to assess the combined effect of CRP and RDW in predicting adverse outcomes in patients with BCNE. CRP > 17.8 mg/L and RDW > 16.3 had significant predictive value for in-hospital death, especially when both factors were combined. In addition, RDW had long-term prognostic value in this patient group, but not CRP.

The prognosis of IE has improved considerably in the past few decades owing to intensive antimicrobial therapy and aggressive surgical intervention. Several risk factors have been identified and used for risk stratification [15–18]. However, in-hospital and long-term mortality remain high, particularly in patients with BCNE. BCNE accounts for 5–69.7% IE cases [19], and is independently associated with postoperative late survival and events [6,20].

Accordingly, identification of patients at moderate or high risk should further improve prognosis. IE is not only an inflammatory entity, but also a condition associated with an autoimmune disorder characterized by elevation of multiple inflammatory markers. Importantly, previous epidemiological studies have reported that increased CRP or RDW level, inflammatory markers, are strongly related to adverse clinical outcomes [11–13] in IE patients. Data from the current study were consistent with these earlier findings.

CRP corresponds to the severity of the underlying disease, while RDW is an index of red blood cell size heterogeneity and it is elevated under conditions of ineffective production and increased destruction of erythrocytes due to inflammatory factors or increased oxidative stress [21, 22]. Therefore, continued increase in CRP and RDW may reflect unfavorable and uncontrollable disease conditions, which expose patients to higher risk of poorer outcome. Increased CRP and RDW, particularly in combination, were independently associated with in-hospital death in the present study. While the combined usage of CRP and RDW did not achieve better performance than RDW alone, its prognostic value was greater (AUC: 0.712 vs. 0.641), signifying that the combination of CRP and RDW could provide a more powerful tool for risk stratification. In clinical practice, patients presenting with BCNE are also at high risk of poor outcomes and may be administered intensive antimicrobial therapy or aggressive surgical intervention in cases with high CRP and RDW.

Our data showed that increased RDW, but not CRP, is associated with high long-term mortality. The underlying mechanisms are yet to be established. The main potential explanation is that CRP is an acute-phase reactant. The level of CRP increases during acute inflammation, which may be suppressed through antimicrobial therapy. However, Lappé *et al.* [23] demonstrated that RDW is associated with chronic inflammation with no significant increase in CRP. In addition, RDW levels may be affected by pathophysiological mechanisms except inflammation, such as oxidative stress [24, 25], facilitating the ability to predict underlying co-morbidities. Therefore, RDW, a simple and inexpensive test, may present a reliable marker for predicting in-hospital and long-term mortality in patients with BCNE.

The present study was a retrospective investigation based on prospective data and had a number of limitations, such as the small sample size from a single center and inclusion of BCNE patients only. Therefore, our conclusions may not be applicable to BCNE patients. Since CRP and RDW were not dynamically monitored, we were unable to ascertain whether marker changes were associated with adverse outcome. Future randomized trials are warranted to confirm whether decreasing CRP and RDW levels below the cut-off points determined in our study would lead to better outcomes.

## MATERIALS AND METHODS

### Patient enrollment and data collection

Patients confirmed with IE according to modified Duke criteria [26] were prospectively enrolled from Guangdong General Hospital, Guangzhou, China, between January 2009 and July 2015. Patients with a history of serious liver dysfunction and hematological disease, except anemia, were excluded. A total of 1293 patients diagnosed with IE were screened, among which 108 were younger than 18 years and 120 were repeatedly hospitalized. Two patients diagnosed as IE after major cardiac surgery during hospitalization, one with missing RDW data, and 155 with no CRP measurements were excluded. We additionally excluded 329 patients with culture-positive IE. In addition, patients with estimated glomerular filtration rate (eGFR) < 15 mL/min/1.73 m^2^ (*n =* 6) were excluded to eliminate interference of serious renal dysfunction, finally resulting in 572 participants. The study was approved by the Research Ethics Committee of Guangdong General Hospital (2016434H). All participants provided written informed consent.

Fasting venous blood was collected after suspected IE, and RDW, CRP and other blood indices measured accordingly. CRP was calculated using an immunonephelometric method (IMMAGE 800, Beckman Coulter, USA; normal range, 0–8 mg/L), and RDW was estimated using an automated blood cell counter (LH780, Beckman Coulter, USA; normal range, 11–16%). Blood was collected from at least three venipuncture sites for cultures, and it was injected into two bottles with specific media for aerobic and anaerobic organisms, respectively. The results were automatically monitored using a blood culture machine. We additionally accepted the results of blood cultures from other centers in cases where patients were transferred to our hospital. Transthoracic M-mode, two-dimensional and Doppler evaluation were routinely performed within 24 h after admission. Left ventricular ejection fraction (LVEF) was determined with the biplane Simpson’s method. Vegetation size was measured in different echocardiographic windows and the longest vegetation diameter obtained. eGFR was calculated using the 4-variable Modification of Diet in Renal Disease equation for Chinese patients [27].

Surgery was performed in cases where surgical criteria were met. However, under conditions of extremely poor health or unacceptably high risk and fee of operation, conservative drug therapy was initiated.

Demographic and clinical characteristics of enrolled participants were collected by one researcher with the aid of an electronic case report form and confirmed independently.

### Follow-up and endpoints

After discharge, all surviving patients were followed up through telephone interviews at least half a year. The primary end point was in-hospital mortality. Secondary end points were long-term all-cause mortality and in-hospital major adverse clinical events (MACEs), including renal dialysis, embolic events, acute heart failure or death. Long-term mortality was defined as all-cause death after discharge to the deadline of follow-up.

### Statistical analysis

In total, 572 participants with BCNE were classified into four groups based on the optimal cut-off values of CRP and RDW determined from receiver operating characteristic analysis: low CRP and low RDW (CRP < 17.8 mg/L and RDW < 16.3%, *n =* 216), high CRP and low RDW (CRP > 17.8 mg/L and RDW < 16.3%, *n =* 129), low CRP and high RDW (CRP < 17.8 mg/L and RDW > 16.3%, *n =* 107), high CRP and high RDW (CRP >17.8 mg/L and RDW >16.3%, *n =* 120). Continuous data were presented as means ± SD and compared using analysis of variance (ANOVA) when normally distributed and otherwise with the Wilcoxon rank-sum test expressed as median and quartile range. Categorical data were presented as a percentage and compared using χ^2^ or Fisher’s exact test. Indices with *P <* 0.05 in univariate analysis were used for multivariate logistic regression analysis, and cut-off points with high sensitivity and specificity confirmed via receiver operator characteristic (ROC) curve analysis. Areas under the curve (AUC) were compared using z statistics [28]. Statistical analyses were performed using SPSS software version 13.0 (SPSS, Inc., Chicago, Illinois). Values of *P* < 0.05 were considered significant.

## CONCLUSIONS

In patients with BCNE, high CRP and RDW, especially in combination, are independently associated with in-hospital death. Early identification of patients with BCNE at higher risk of in-hospital complications using the combination of CRP and RDW may improve therapeutic choices and consequent outcomes. Furthermore, RDW has long-term prognostic value in patients with BCNE, but not CRP.

## Abbreviations

CRP: C-reactive protein
RDW: red cell distribution width
BCNE: blood culture-negative infective endocarditis
OR: odds ratio
CI: confidence interval
IE: infective endocarditis
eGFR: estimated glomerular filtration rate
LVEF: left ventricular ejection fraction
MACEs: major adverse clinical events
NYHA: New York Heart Association
ESR: erythrocyte sedimentation rate
WBC: white blood count
